# Supplementary material for: Genome-Wide Identification and Comprehensive Analysis of the FtsH Gene Family in Soybean (Glycine max)
Source: Int J Mol Sci. 2023 Nov 30;24(23):16996. doi: 10.3390/ijms242316996 (PMC10707429; doi:10.3390/ijms242316996)
Supplement: Supplementary file 1 [file ijms-24-16996-s001.zip › Figure S1.pdf]

Group I

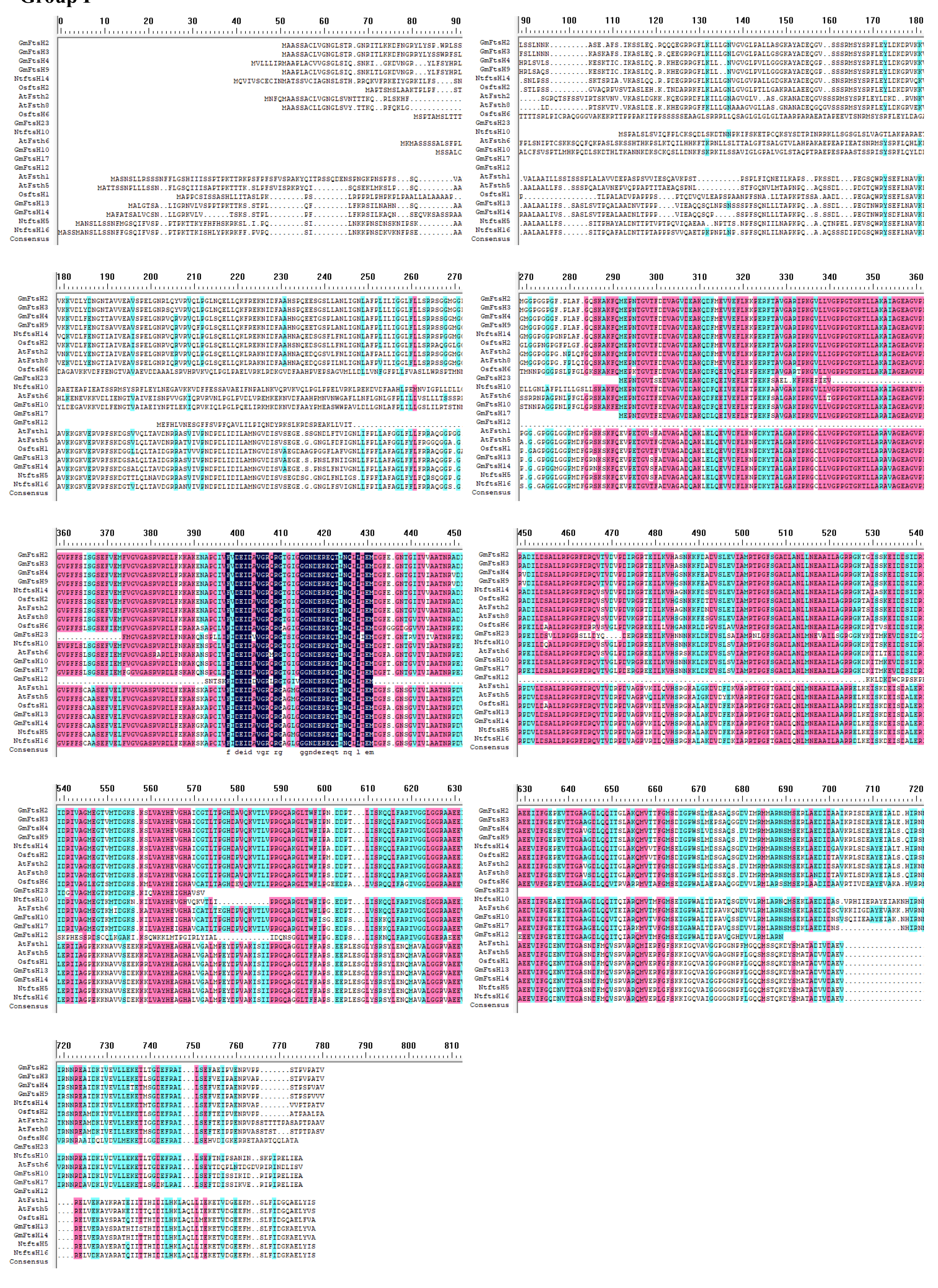

Group II

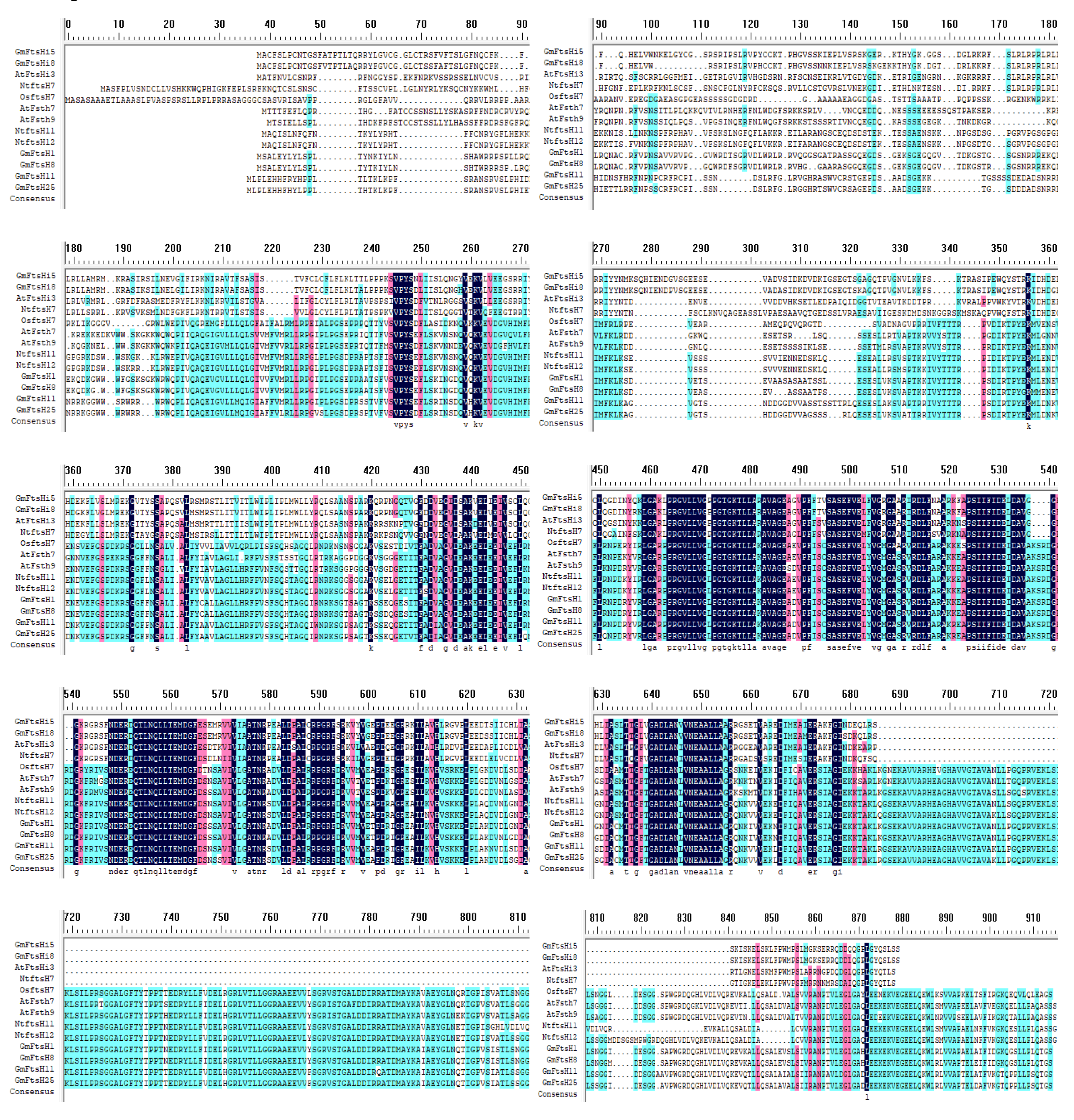

Group III

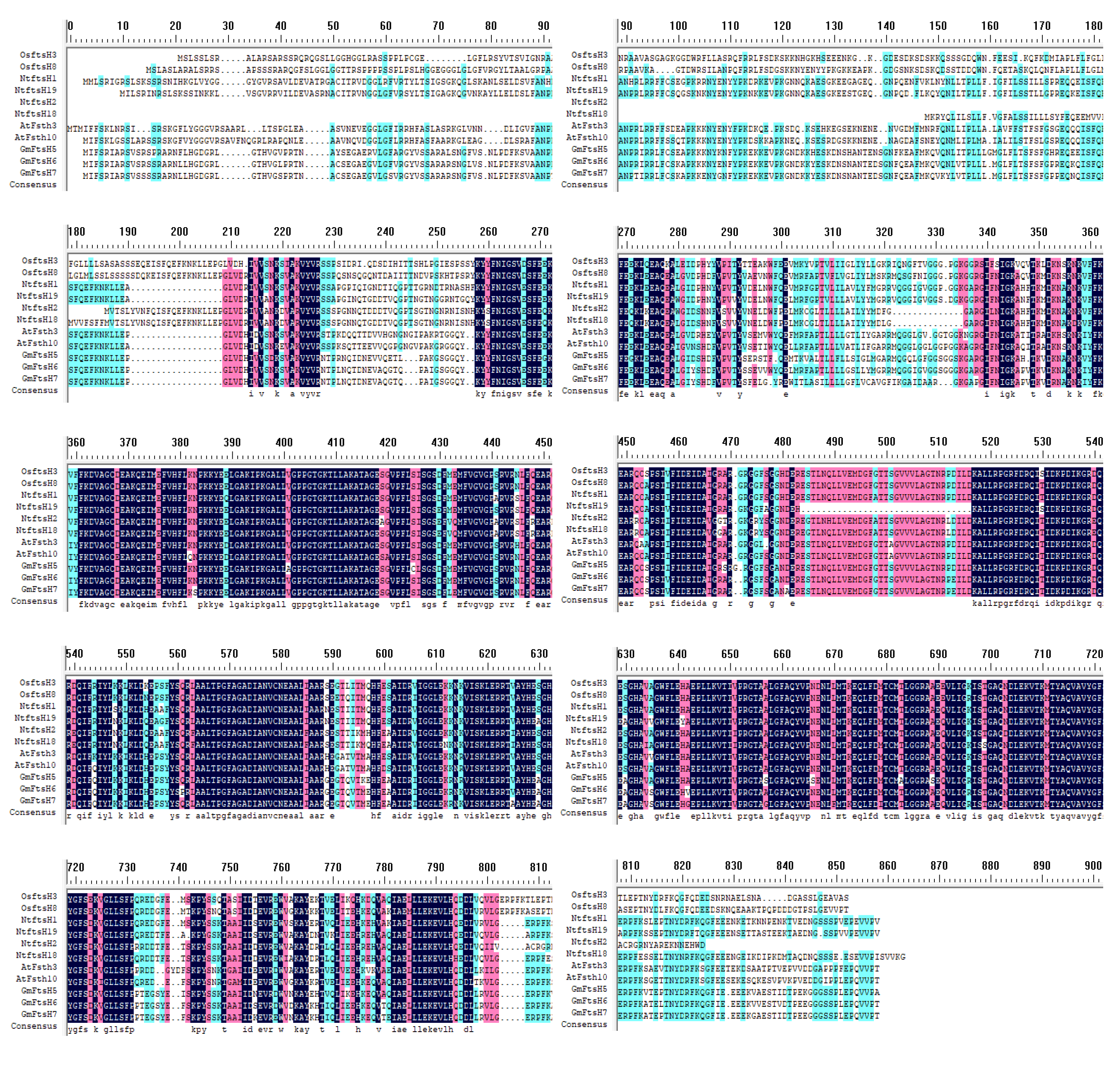

Group IV

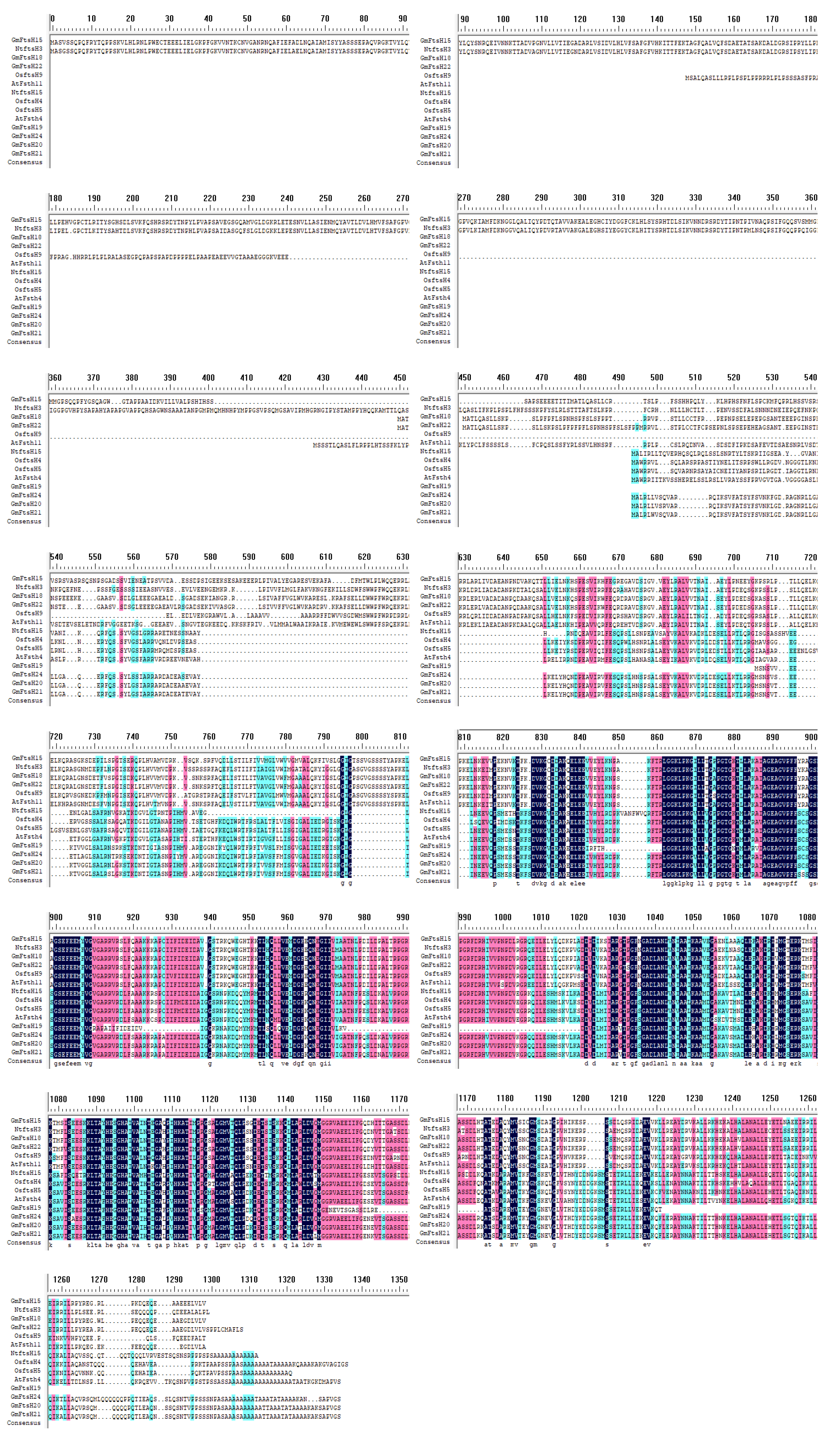

Group V

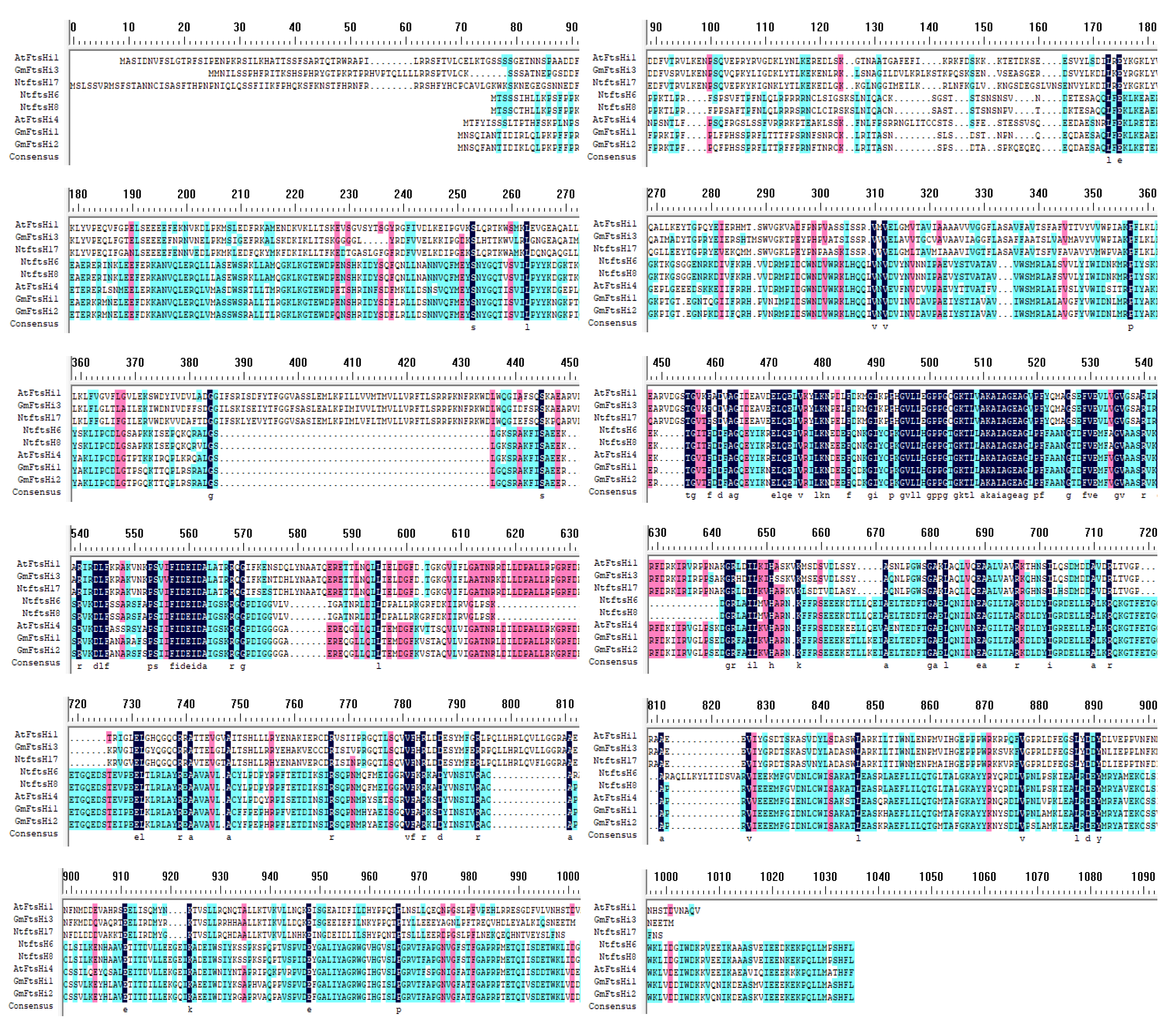

Group VI

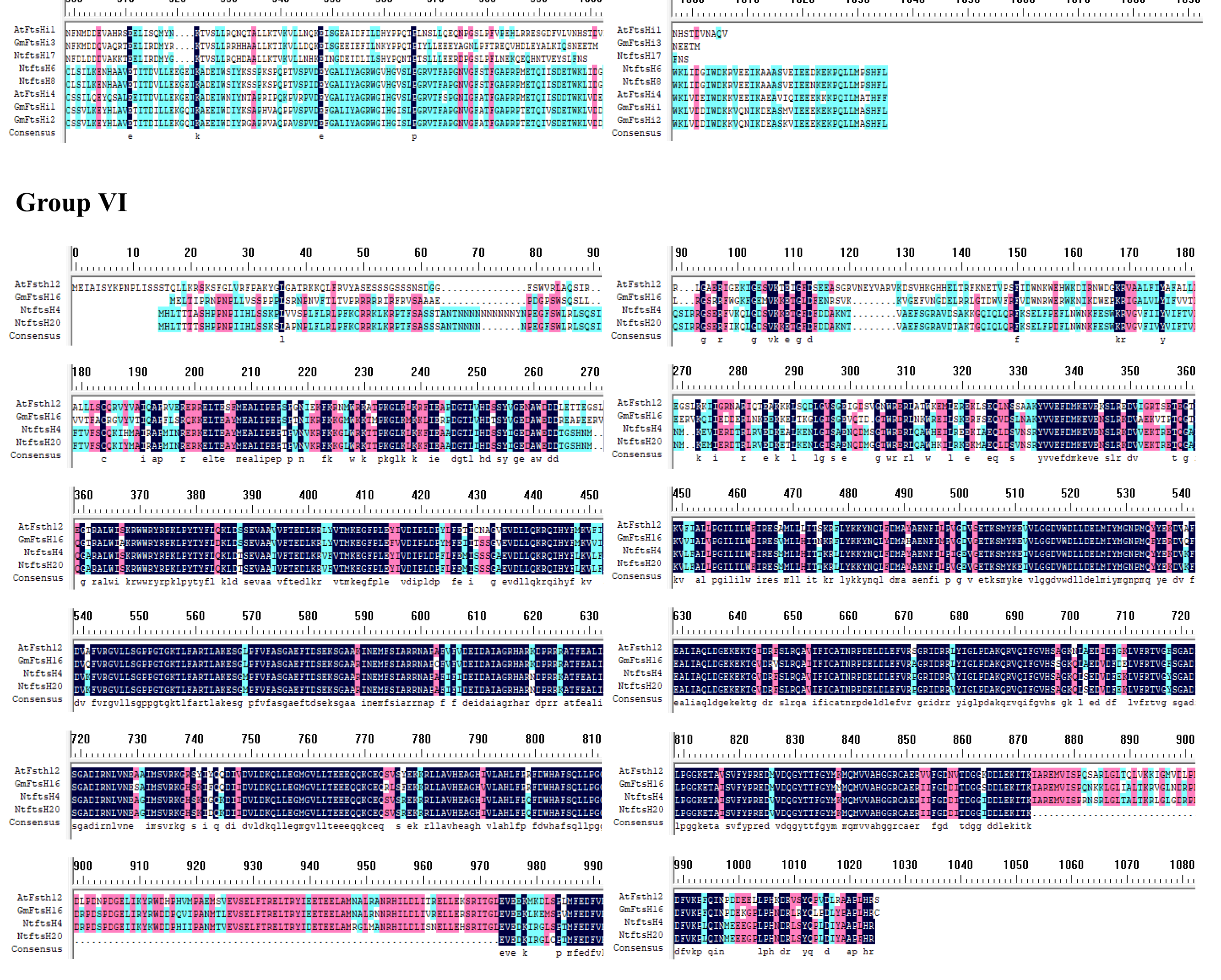

Group VII

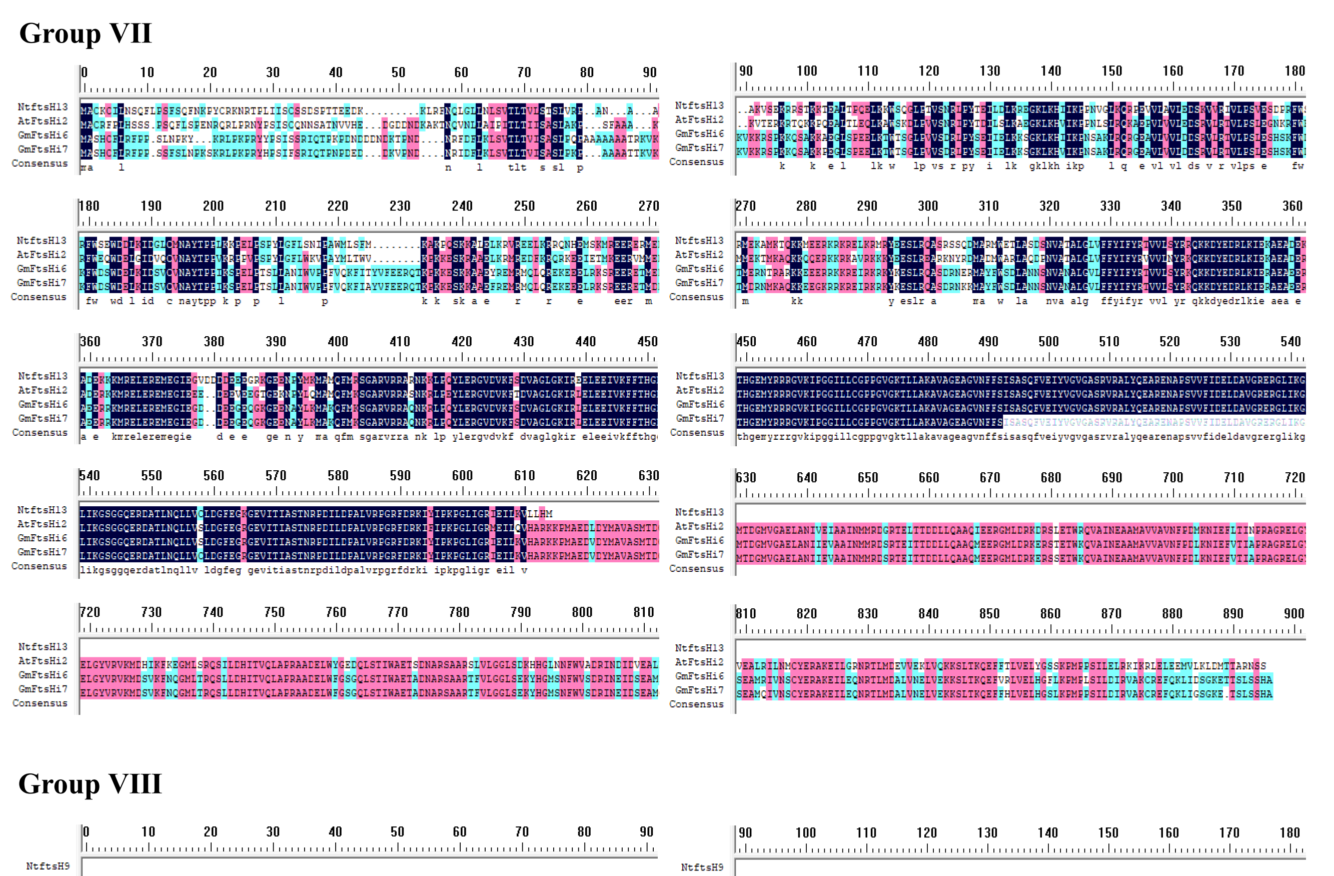

Group VIII

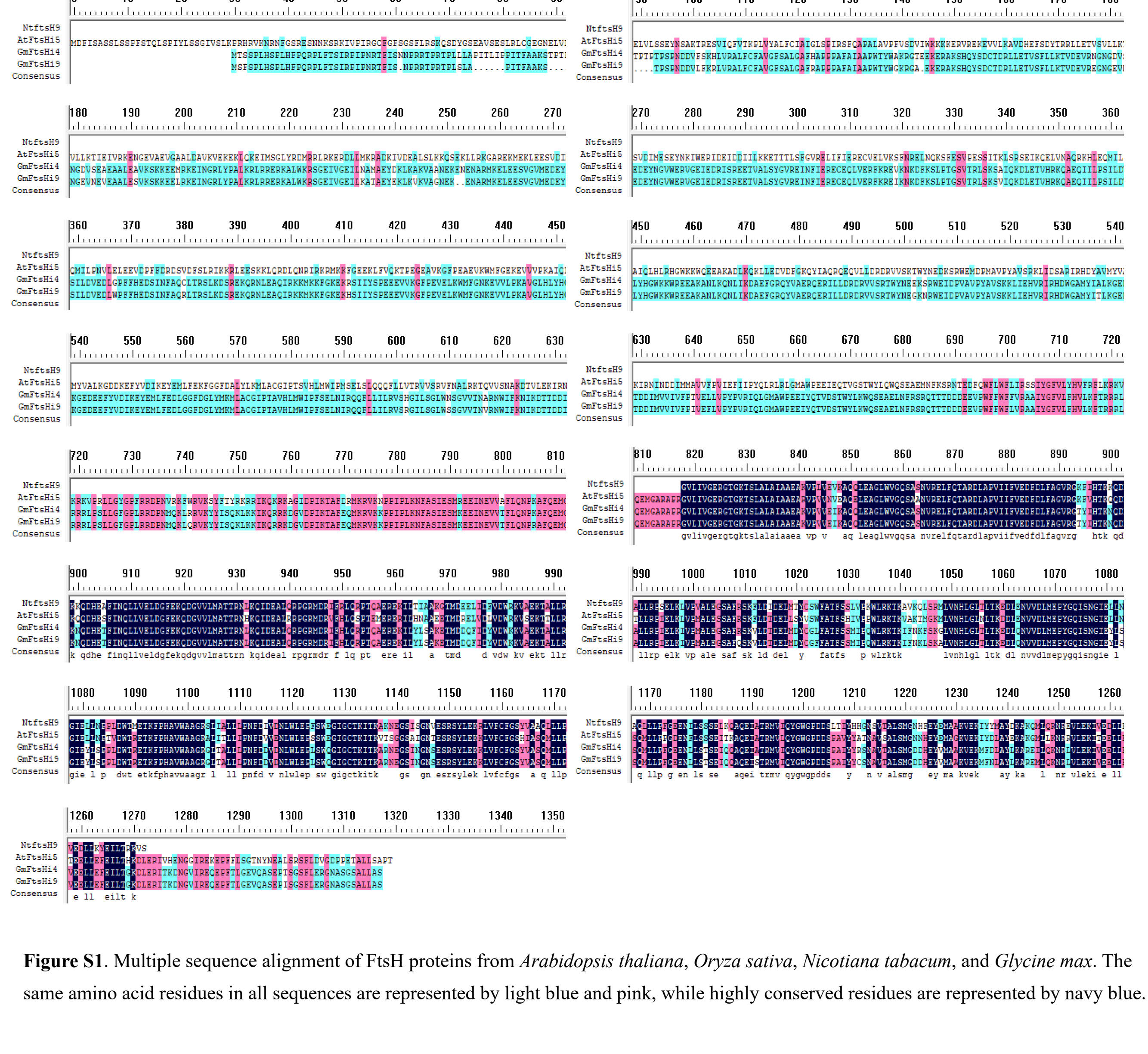

Figure S1. Multiple sequence alignment of FtsH proteins from *Arabidopsis thaliana*, *Oryza sativa*, *Nicotiana tabacum*, and *Glycine max*. The same amino acid residues in all sequences are represented by light blue and pink, while highly conserved residues are represented by navy blue.
