# Supplementary material for: Genome-Wide Identification and Comprehensive Analysis of the FtsH Gene Family in Soybean (Glycine max)
Source: Int J Mol Sci. 2023 Nov 30;24(23):16996. doi: 10.3390/ijms242316996 (PMC10707429; doi:10.3390/ijms242316996)
Supplement: Supplementary file 1 [file ijms-24-16996-s001.zip › Figure S2.pdf]

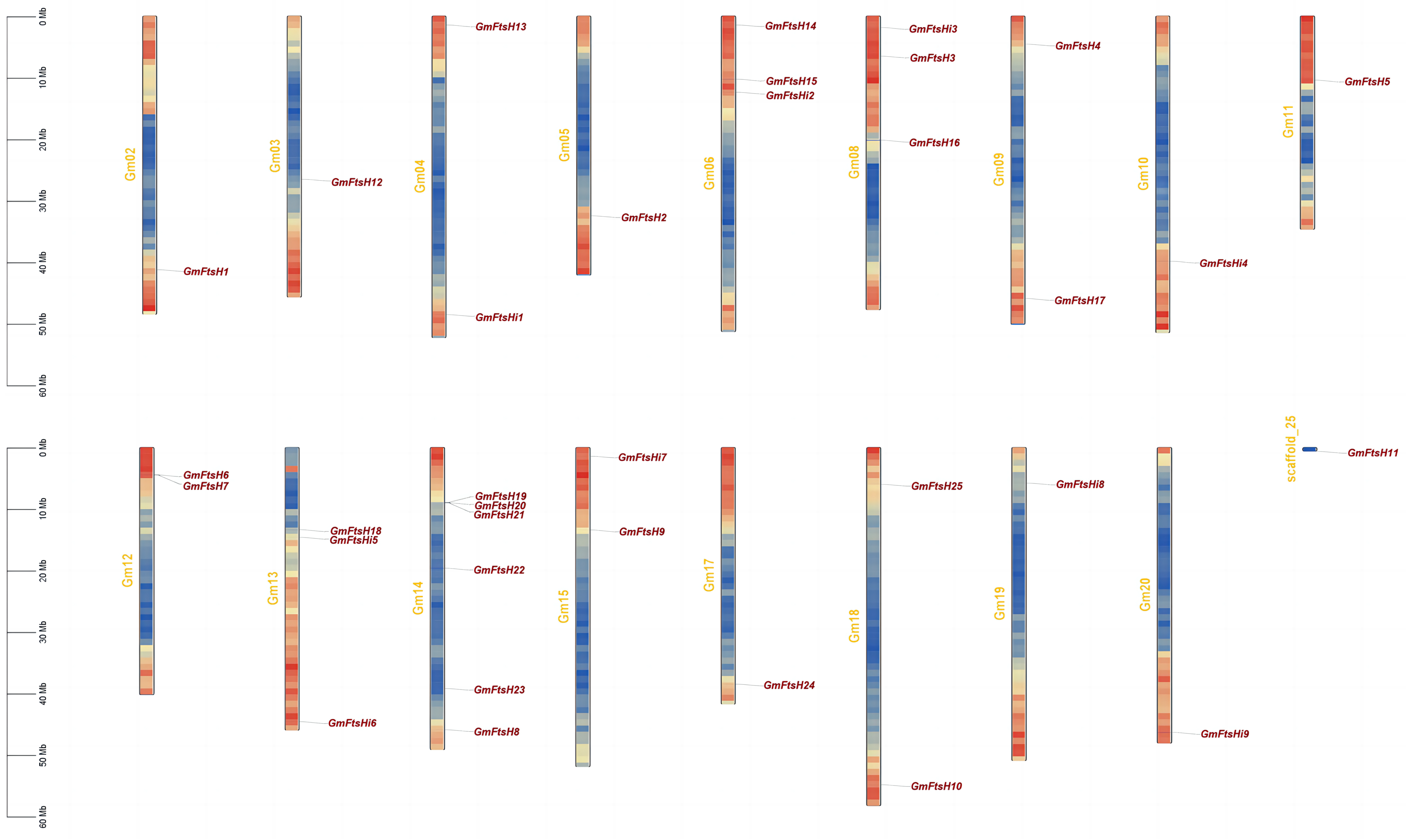

Figure S2. Chromosomal location analysis of *GmFtsH* genes. Gm01~Gm20 represent 20 chromosomes of *Glycine max*. The scale on the left shows megabases (Mb).
