# Supplementary material for: Genome-Wide Identification and Comprehensive Analysis of the FtsH Gene Family in Soybean (Glycine max)
Source: Int J Mol Sci. 2023 Nov 30;24(23):16996. doi: 10.3390/ijms242316996 (PMC10707429; doi:10.3390/ijms242316996)
Supplement: Supplementary file 1 [file ijms-24-16996-s001.zip › Table S4.pdf]

Supplementary Table S4. List of Primers

Primer name	Primer sequence
GmFtsH1-RT-F	CTGCTGCTACTTCTTCTTTGGAG
GmFtsH1-RT-R	TCCACCTCATTTTCCAGCATCT
GmFtsH2-RT-F	CCGAGTTGGGGAATCGGTTG
GmFtsH2-RT-R	AAGCAAGAGGAAATCCGGGC
GmFtsH5-RT-F	ATGTAAGGAACACTCCCCGC
GmFtsH5-RT-R	AGAGCAACCTTCGTCATTTCC
GmFtsH10-RT-F	CCCACTTTGATGCACAAGCC
GmFtsH10-RT-R	TTCTGGCTCAGCTCTTGTGG
GmFtsH11-RT-F	ACTACTAGGTTGCAGGAATCAGA
GmFtsH11-RT-R	TGGGGAACCAAACTCCACCT
GmFtsH12-RT-F	CAGCAGAGAAGCCAAACTCCT
GmFtsH12-RT-R	CTGGCAAGAATCACGGCTTG
GmFtsH13-RT-F	GCCTTGATCGGAAGGAATGT
GmFtsH13-RT-R	GCAGCTTGTGAATTGTGAGCA
GmFtsH16-RT-F	GCTTGAGGGTATGGGTGTCC
GmFtsH16-RT-R	GGCAGGAGCTGTGAAAATGC
GmFtsH24-RT-F	GGCGTACACTTAGGTTCATTGCAG
GmFtsH24-RT-R	GCTCTTCCTTTGCCTCATCAAC
GmFtsHi1-RT-F	CTCACCACCACATTCCCCTC
GmFtsHi1-RT-R	TTCATCCGTTTCCTCTCCGC
GmFtsHi4-RT-F	CCACGAACCCCACTCTTACTC
GmFtsHi4-RT-R	GGCTCTCTCTTTCTCCTCGG
GmFtsHi5-RT-F	TTTGGGTGTGTGTGGAGGTT
GmFtsHi5-RT-R	CCCTAGCTCCTTGTTCCACAC
GmFtsHi6-RT-F	AAAAGACTTCCCAAACCCCGT
GmFtsHi6-RT-R	GGGGTTTTGTCATTGTCGTCG
GmFtsH18-RT-F	TCCACGCTCCTATGTTGCAC
GmFtsH18-RT-R	GCACCTTCTTTTTCTTCTTCGGG
GmActin11-RT-F	GCGGGAAATTGTAAGGGATGT
GmActin11-RT-R	TCGCCAATAGTGATGACCTG
GmFtsH13-F	ATGGCCTTGGGCACCAGCGCCTT
GmFtsH13-R	TTATGCAACATATAATTCGGC
pGDG-GmFtsH13-F	TCTCTCTACAAGATCTCGAGATGGCCTTGGGCACCAGCGC
PGDG-GmFtsH13-R	CCCTTGCTCACCATGTCGACTGCAACATATAATTCGGCTT
